# Functional analysis of a CDKN2A 5’UTR germline variant associated with pancreatic cancer development

**DOI:** 10.1371/journal.pone.0189123

**Published:** 2017-12-07

**Authors:** William Bruno, Virginia Andreotti, Alessandra Bisio, Lorenza Pastorino, Giuseppe Fornarini, Stefania Sciallero, Giovanna Bianchi-Scarrà, Alberto Inga, Paola Ghiorzo

**Affiliations:** 1 Genetics of Rare Cancers, Department of Internal Medicine and Medical Specialties (DiMI), University of Genoa and Ospedale Policlinico San Martino, Genoa, Italy; 2 Centre for Integrative Biology (CIBIO) and University of Trento, Trento, Italy; 3 Medical Oncology Unit, Ospedale Policlinico San Martino, Genoa, Italy; CNR, ITALY

## Abstract

*CDKN2A* coding region germline variants are associated with pancreatic adenocarcinoma (PC) susceptibility. Recently, we described functional germline 5’UTR *CDKN2A* variants from melanoma patients affecting the post-transcriptional regulation of p16^INK4a^ mRNA that is dependent, at least in part, on an Internal Ribosome Entry Site (IRES) in the 5’UTR region. Here we describe a 5’UTR c.-201_-198delinsCTTT *CDKN2A* variant (frequency 0.0028 based on 350 PC patients), which seems to be private to PC, since it has never been found in public databases nor in thousands of melanoma patients tested. Functional analyses confirmed IRES activity of the 5’UTR in BX-PC3 PC cells and revealed a functional impact of the identified variant. Using gene reporter assays we observed reduced translation potential in cells treated with the mTOR inhibitor Torin1, a condition that favors the assessment of IRES activity. At the endogenous gene level we quantified allelic imbalance among polysome-associated mRNAs using a patient-derived cell line heterozygous for the c.-201_-198delinsCTTT. Overall, we conclude that this very rare private variant can be considered a potential mutation, specifically associated with PC. Our data indicate that sequencing of the entire 5'UTR of *CDKN2A* should be included in routine screening of PC cases with suspected inherited susceptibility.

## Introduction

Pancreatic adenocarcinoma (PC) is a fatal cancer, with rapid progression and a high death rate. The majority of patients die within a year of diagnosis. One of the best strategies to reduce the mortality of the disease is to improve early diagnosis; therefore, it is important to identify individuals at high risk as candidates for surveillance protocols. Familial inheritance is implicated in approximately 10% of PC cases. Although genetic predisposition to PC remains largely unexplained, next-generation sequencing has led to the new identification of mutations in candidate genes. Among them, high penetrance mutations in *BRCA1*, *BRCA2*, *CDKN2A*, *STK11/LKB*, *ATM*, *PALB2*, and DNA mismatch repair genes, usually in the context of familial cancer syndromes, have been described [1,2].

The *CDKN2A* gene plays a key role in PC etiology. It is one of the most commonly somatically mutated genes in PC, and *CDKN2A* promoter hyper-methylation has been suggested to play a critical role in PC development [3]. Germline mutations in the *CDKN2A* gene are typically found in 20 to 40% of melanoma families, which may also have an increased incidence of PC [4–13].

However, although almost all pancreatic adenocarcinomas have inactivating alterations of this gene, until recently *CDKN2A* was not considered a PC susceptibility gene outside of the known association with melanoma and the melanoma-PC syndromes. In the last few years, rare germline mutations have been associated with increased risk of developing pure familial PC and several studies showed differing percentages of germline mutations in familial PC cases [1,2]. In Italy, germline mutations in the *CDKN2A* coding region account for a considerable percentage of familial PC cases (FPC) [14].

We recently performed functional studies of 5’UTR *CDKN2A* germline mutations in melanoma patients and identified an Internal Ribosomal Entry Site (IRES)-like activity in the 5’UTR of *CDKN2A* [15–17]. Further, we identified a variant of unknown functional significance in the 5’UTR of an FPC patient that we had never found in melanoma cases [14] and we hypothesized that a subset of PC patients could harbor rare functional variants in the 5’UTR of *CDKN2A* gene as well.

This hypothesis required experimental validation both *in vitro* by functional studies, and in the clinical setting to establish the frequency of these variants in a broad cohort of PC patients. Therefore aim of this study was to further investigate the *CDKN2A* 5’UTR in an extended cohort of PC patients and use the previously proposed pipeline for functional analysis, including gene reporter assays, polysomal profiling and western blot, as a further contribution towards establishing the effect of these 5’UTR variants on cap-independent translation when the cap dependent process is inhibited.

## Materials and methods

### PC cases and controls

Three hundred and fifty PC patients and an equal number of healthy controls were selected from an ongoing case-control study being conducted at our local hospital. All the PC diagnoses were confirmed by histological or medical records. Both patients and controls signed an informed consent form, provided a blood sample and answered a questionnaire with the aid of a trained interviewer, following local Ethics Committee-approved protocol (CE IRCCS San Martino–IST Istituto Nazionale per la Ricerca sul cancro, Genova, Italy, EDG98.001).

### Sequencing of the *CDKN2A* 5’UTR

We analyzed the 5’UTR of *CDKN2A* (NM000077.4) by sequencing up to the 260^th^ base upstream of the ATG in all patients and controls as previously described. Standard primers and procedures for sequencing, which have been previously described, were adopted [5,18,19]. 5’UTR sequencing data from a cohort of 1,650 melanoma cases were also evaluated [14,16].

### Gene reporter assays and cap-dependent inhibition

Briefly, the BX-PC3 human primary pancreatic adenocarcinoma cell line was transfected with pGL3-promoter and pRuF bicistronic vectors containing the whole wild-type (wt) or variant *CDKN2A* 5’UTR as described in detail described [16]. The same assay was performed in WM266-4, G361, SK-MEL-5 metastatic melanoma cell lines as a control for the possible effect on melanoma cell lines.

The MCF7 human breast adenocarcinoma cell line was also transfected with the bicistronic reporter and treated with the mTOR inhibitor Torin1 50nM for 16 hours [17]. All these cell lines were null for p16 ^INK4a^. HPNE normal ductal pancreatic cell line were transfected with Viafect (Promega) as suggested by the manufacturer and treated with the mTOR inhibitor Torin1 50nM for 16 hours. Each transfection experiment was performed with triplicates and the transfection was repeated 3 times. Results are presented as average relative light units and values obtained with 5’UTR variants were compared with those obtained with the wild type using t-test.

### mRNA quantification and protein expression in patient-derived lymphoblastoid cell lines (LCLs)

qPCR measurements of endogenous *CDKN2A* mRNA, and protein quantification was performed by Western Blot as previously described [16].

A lymphoblastoid cell line was derived from the patients harboring the CDKN2A 5’UTR variant in the heterozygous state and from a control wt for the 5’UTR and total RNA was extracted, after which the relative expression of the total RNA was measured by qPCR. The average normalized expression of p16 ^INK4a^ and the standard deviation of three replicates are presented. Soluble proteins were recovered from total cell extracts from the pair of cell lines, and relative expression of p16 ^INK4a^ was quantified by western blot.

### Polysomal allelic imbalance

The relative abundance of the two 5’UTR polysomal alleles at endogenous level was evaluated by Next Generation Sequencing using an Ion PGM^TM^ System (Life Technologies), and confirmed by Sanger sequencing, starting from patient and control LCLs as described in detail previously [16,17].

## Results

### The c.-201_-198delinsCTTT germline *CDKN2A* 5’UTR variant is present in PC patients and absent in melanoma cases

**Fig 1:**
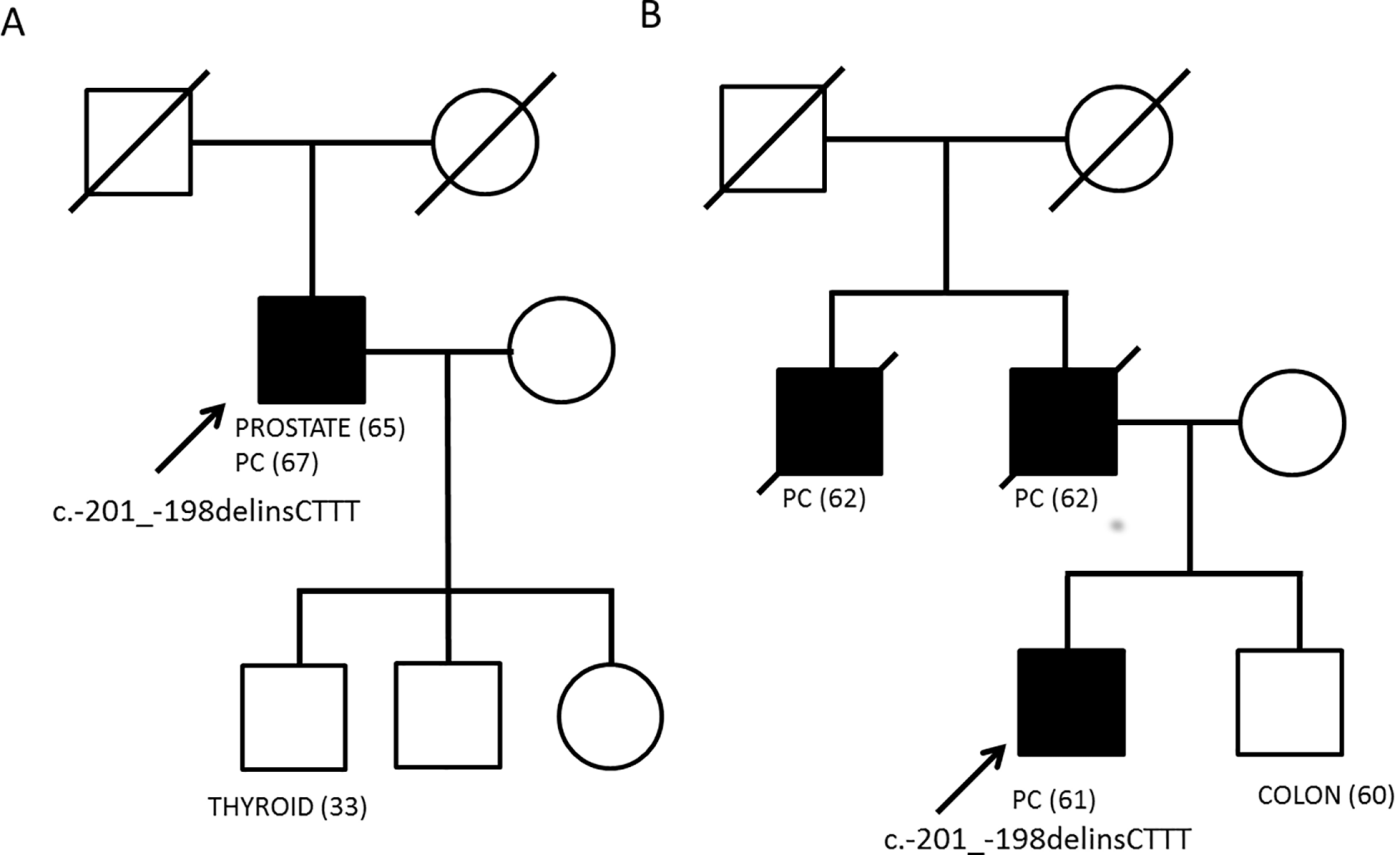
Pedigrees of PC patients carrying the *CDKN2A* 5’UTR c.-201_-198delinsCTTT germline variant. Age at cancer diagnosis and type of cancer is provided below each symbol. Pancreatic adenocarcinoma (PC) is indicated with black symbols and the probands are indicated by an arrow.

Two out of 350 PC cases were found to harbor the c.-201_-198delinsCTTT germline variant (frequency = 0.0028). The two cases were unrelated and were negative for germline mutations in the coding region of *CDKN2A*. One was a sporadic PC case, also affected by prostate cancer while the other was a pure familial case having two first-degree relatives affected by PC. Co-segregation analysis was not possible due to lack of DNA from the deceased affected relatives (Fig 1). To our knowledge this variant has never been described in PC patients, nor in public databases (Ensembl, Exac, LOVD, ClinVar, Gnomad). The variant was not found in a cohort of 350 healthy population/regional controls and in our selection of 1,863 melanoma cases.

### The c.-201_-198delinsCTTT variant allele exhibits reduced translation potential in cells treated with Torin1

**Fig 2:**
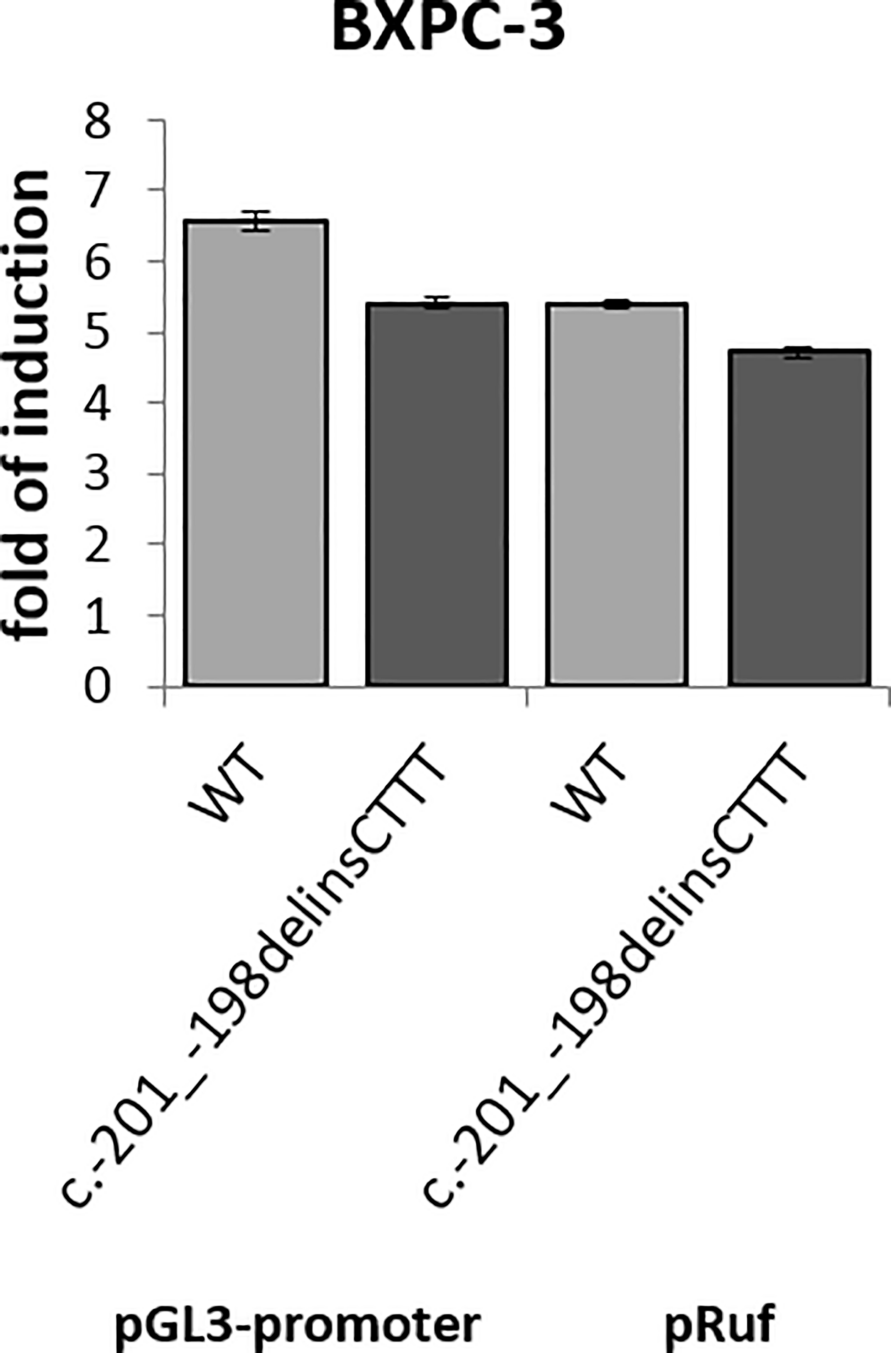
No impact of the *CDKN2A* 5’UTR c.-201_-198delinsCTTT variant was observed in the pGL3-promoter and in the bicistronic pRuf reporter vector as compared to the wt. The assay was performed in the pancreatic cell line BXPC-3 null for p16 ^INK4a^ protein.

Dual luciferase assays were conducted after transient transfection in BX-PC3 PC cell line that are null for p16 ^INK4a^ protein expression (Fig 2). First we transfected the pGL3-promoter vector containing the wt and variant 5’UTR upstream of the firefly cDNA start codon and immediately downstream of the viral promoter SV40. Acting as the 5’UTR of the firefly luciferase, this construct could help to rule out the possibility of effects on transcriptional activity, which we previously excluded for 5’UTR single nucleotide variants (SNV) identified in melanoma cases. As expected we did not observe any differences in luciferase activity between pGL3-promoter vector wt and c.-201_-198delinsCTTT variant. Both alleles resulted in a comparable induction of the reporter activity compared to the empty vector.

We then used the bicistronic vector with the 5’UTR wt or variant cloned between the Renilla reniformis and the Firefly luciferase and compared the relative activities of the two luciferase enzymes which are produced as a part of a single transcript. In the pRuF bicistronic vector, the Renilla reporter is produced by cap-dependent translation, while the Firefly (one) originates from ribosome loading occurring at the *CDKN2A* 5’UTR sequence, acting as an IRES.

Transfection of the wt 5’UTR in BX-PC3 PC cells confirmed IRES activity. However, unlike our past observations with the same experiment on SNVs in melanoma cases, which allowed us to identify the 5’UTR working as an IRES to modulate p16 ^INK4a^ translation, we did not see any differential impact on cap-independent translation of the Firefly reporter with variant 5’UTR (Fig 2). The same analysis, with wt and c.-201_-198delinsCTTT variant 5’UTR was performed in p16 ^INK4a^ null melanoma cell lines (WM266-4, G361 and SK-MEL-5) and no differences were observed.

**Fig 3:**
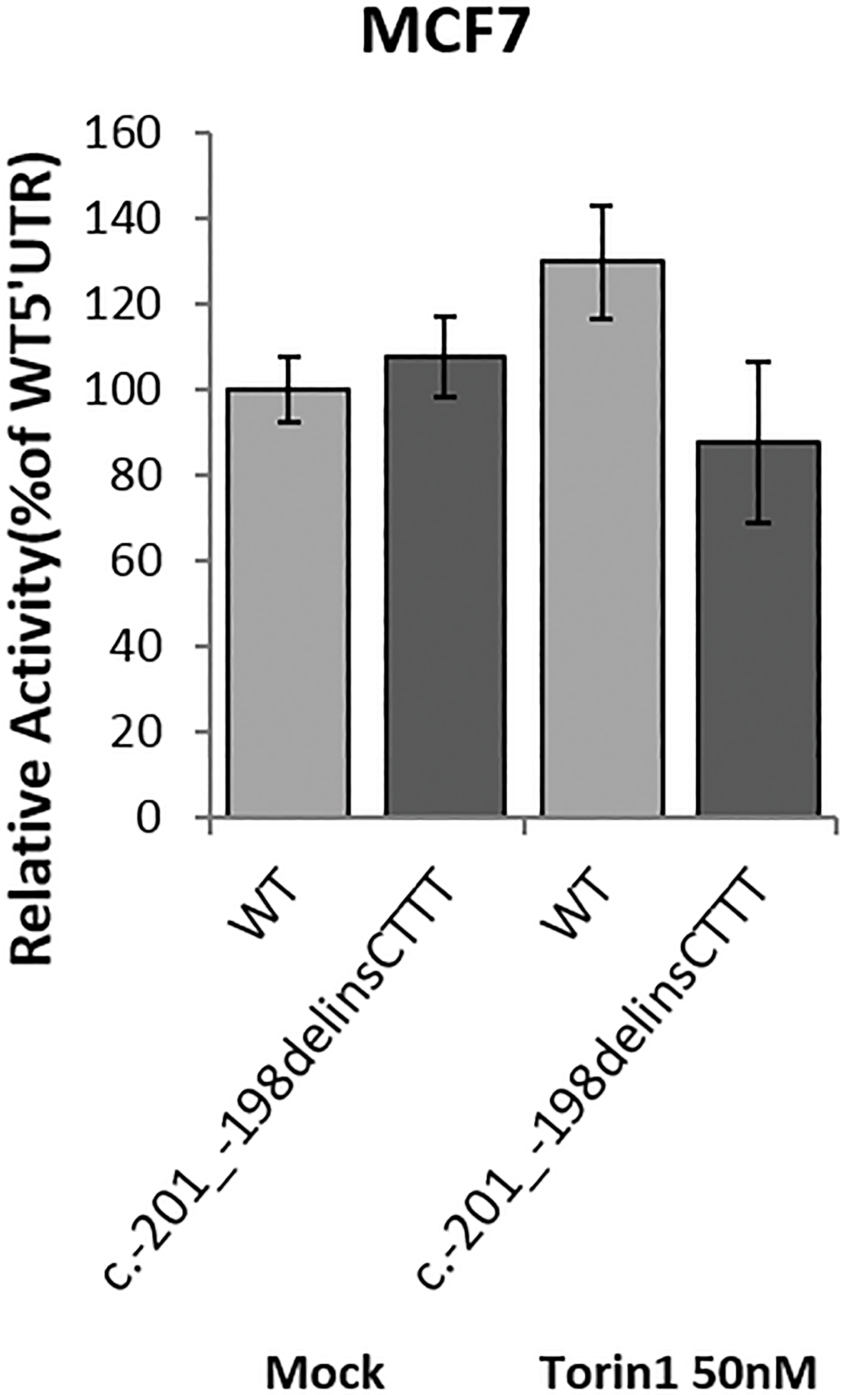
The c.-201_-198delinsCTTT variant allele exhibits reduced translation potential in MCF7 cells treated with Torin1.

We then transfected the MCF7 cells with the same wt and variant bicistronic vector, in standard conditions, and treated them with the mTOR inhibitor Torin1 to inhibit the cap dependent translation in order to reveal a subtle effect which may have gone undetected without the treatment, as previously suggested [17]. The MCF7 cells were chosen for this experiment because we had already set up conditions for transfections and dose treatment in our previous study identifying the IRES activity of the 5’UTR (Fig 3). In addition MCF7 cells had previously been used in the luciferase assay with wt and 5’UTR SNVs and no differences were observed in comparison with the melanoma cell lines like WM266-4,G361,SK-MEL-5 [16].

**Fig 4:**
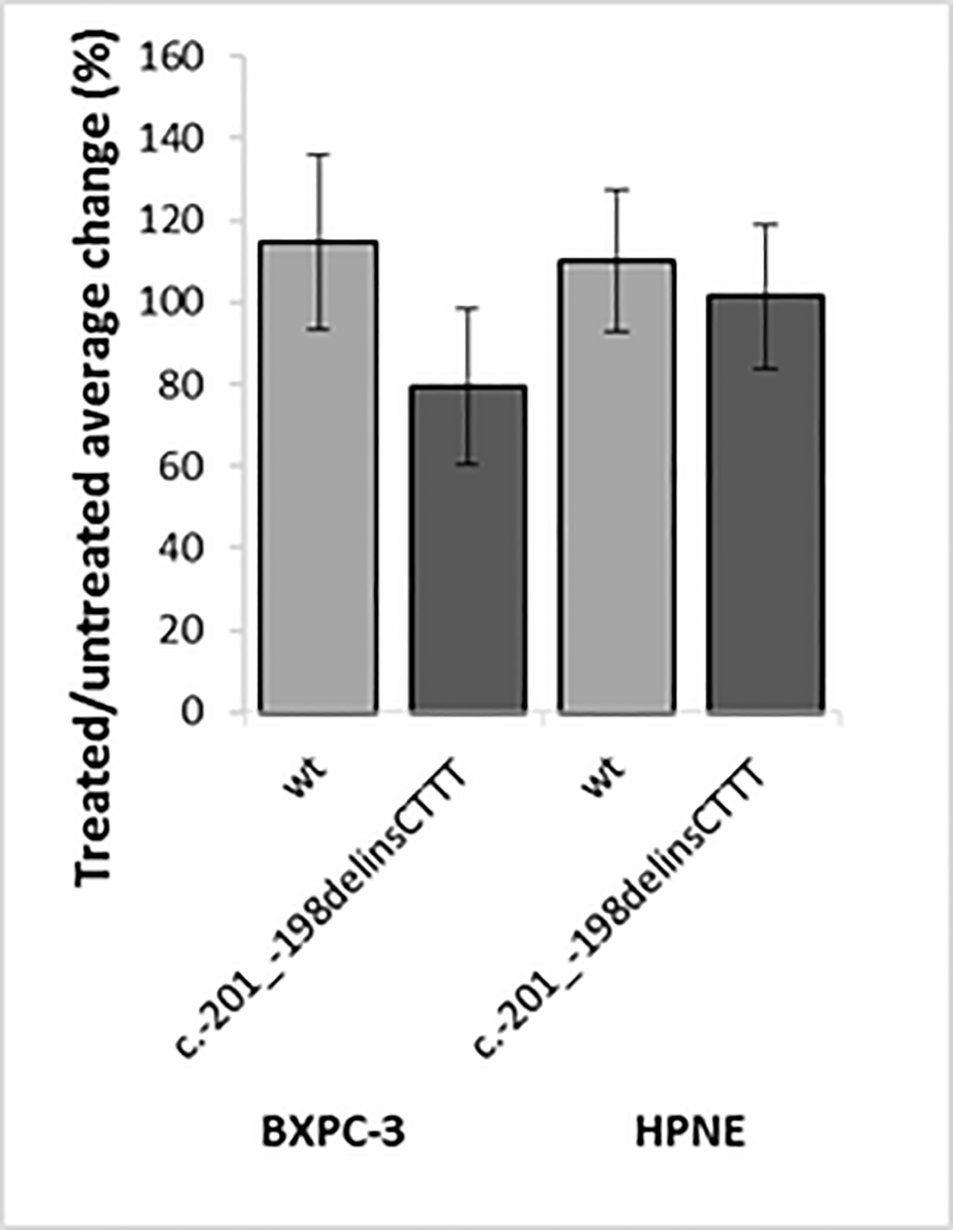
The c.-201_-198delinsCTTT variant allele (treated/ untreated) exhibits reduced translation potential in BXPC-3 cells treated with Torin1 compared to the wild type, while a non significant trend was apparent also in normal ductal adenocarcinoma cells (HPNE). The activity of each plasmid in untreated condition was set to 100%.

While in untreated cells the c.-201_-198delinsCTTT variant behaved like the wt 5’UTR, thus confirming the results in the pancreatic and melanoma-derived cell lines, treatment with 50nM Torin1 for 16 hours led to the expected increase in the relative reporter activity for the wt 5’UTR but to a decrease for the variant 5’UTR (p<0.05, t-test), suggesting a subtle defect of the c.-201_-198delinsCTTT variant allele activity in conditions where cap-dependent translation is partially inhibited. The same analysis was extended to BX-PC3 and HPNE normal ductal pancreatic cells. For these experiments we compared using the bicistronic pRuF plasmid the p16ink4a wild type 5’UTR and the c.-201 variant. Similarly to the results obtained in MCF7 cells the c.-201 variant did not reduce the relative activity of the downstream Firefly reporter in all cell lines tested. However, the treatment with Torin1 led to a reduction in the activity of the cap-independent reporter which was significantly stronger for the c.-201 variant compared to the wild type in BXPC3 cells (t-test = 0.03). A non significant trend was apparent also in HPNE (t-test = 0.58) (Fig 4).

Overall, functional analyses confirmed IRES activity of the 5’UTR in BX-PC3 PC cells and revealed a functional impact of the identified variant.

### Allelic imbalance and inhibition of cap-dependent translation in lymphoblastoid cell lines from patients carrying the c.-201_-198delinsCTTT 5`UTR variant

**Fig 5:**
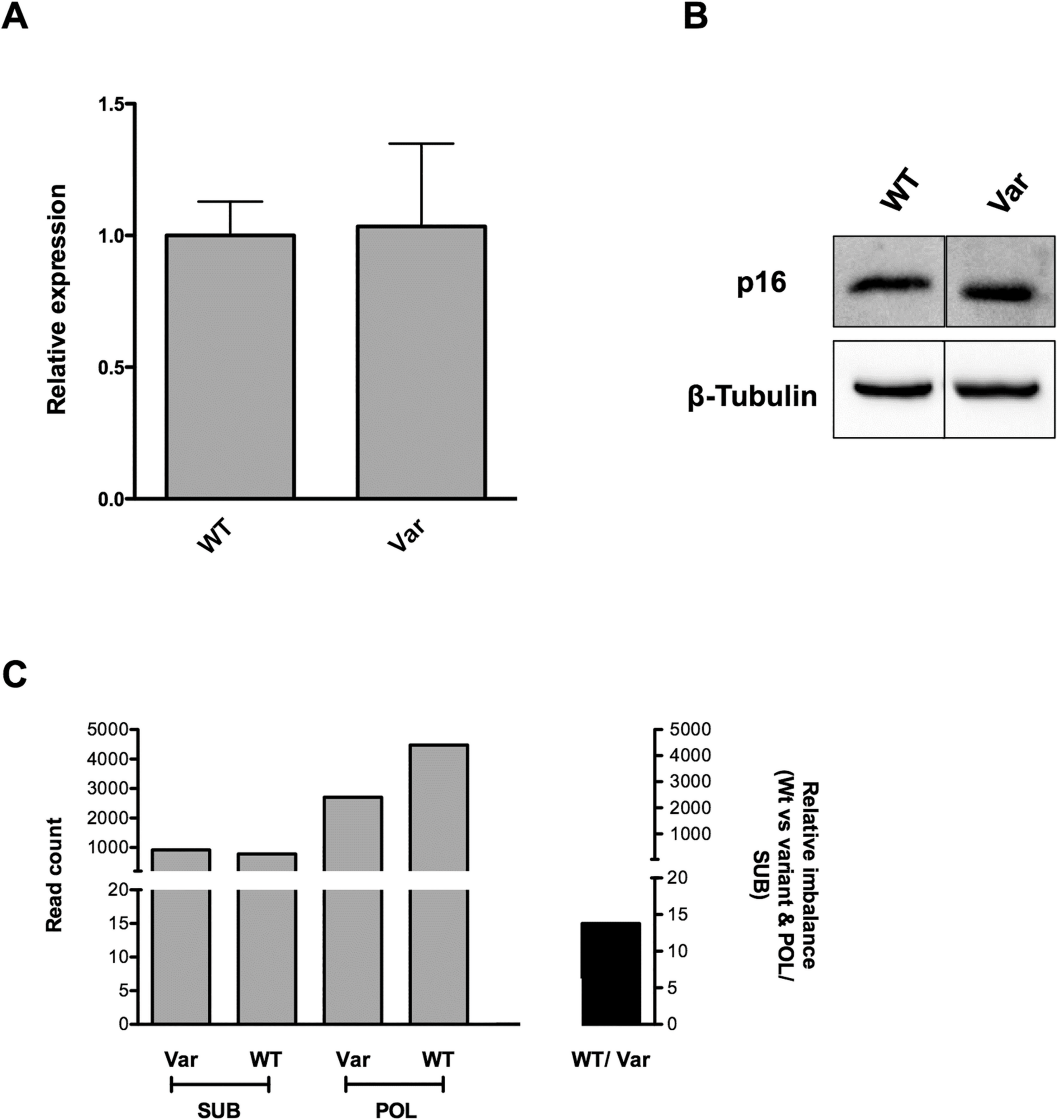
Relative p16 ^INK4a^ mRNA and protein expression in c.-201_-198delinsCTTT variant, measured by qPCR (A) and Western Blot (B); for qPCR, the average normalized expression of p16^INK4a^ and the standard deviation of three replicates are presented. (C) Polysomal c.-201_-198delinsCTTT allelic imbalance. Cytoplasmic lysates of the lymphoblastoid cell line heterozygous for the c.-201_-198delinsCTTT variant were fractionated using 15–50% sucrose gradients and RNAs co-sedimenting with subpolysomal fractions or with polysomes were extracted and used to amplify and sequence the p16^INK4a^ 5’UTR by Ion Torrent. The Y-axis on the left displays the allelic coverage, while the one on the right plots the percentage of relative imbalance of the wt allele in the comparison between polysomal and subpolysomal fractions. SUB = subpolysomal; POL = polysomal; Var = c.-201_-198delinsCTTT variant.

We also evaluated the effect of the *CDKN2A* 5’UTR variant on mRNA and protein expression at the endogenous level. Using qPCR and western blot we measured p16^INK4a^ mRNA and protein relative expression starting from a p16 ^INK4a^ wt lymphoblastoid cell line and from one lymphoblastoid line heterozygous for the c.-201_-198delinsCTTT variant. No significant differences were observed (Fig 5A and 5B).

Although no impact of the c.-201_-198delinsCTTT change was appreciable on steady-state mRNA or protein levels in the heterozygous cell line, allele-specific tests were developed to more precisely quantify their relative expression and association with the polysomes (Fig 5C).

Ion Torrent-based sequencing of p16 ^INK4a^ mRNA was performed after sucrose-gradient fractionation of cytoplasmic mRNA fractions recovered from the c.-201_-198delinsCTTT heterozygous cell line. We compared the subpolysomal (free, 40S, 60S and 80S) fractions, corresponding to mRNA not undergoing translation to the polysomal (2 or more ribosomes) fractions, corresponding to actively translated mRNA and quantified the relative expression of the two alleles by read count. Results were confirmed by Sanger sequencing through quantification of the electropherograms.

In conclusion the variant allele c.-201_-198delinsCTTT shows no obvious transcriptional or stability alteration, but we can observe a reduced association to the polysome, which may lead to a slight reduction in protein expression that may contribute to increased, tissue specific cancer risk.

## Discussion

In this study we performed functional testing of a rare private germline *CDKN2A* 5’UTR c.-201_-198delinsCTTT variant specifically associated with PC which we classified as a potential mutation, after applying a pipeline for functional analysis of 5’UTR variants we recently proposed studying *CDKN2A* 5’UTR variants in melanoma patients [16,17].

In those studies we identified 17 germline SNVs in the *CDKN2A* 5’UTR in melanoma patients during routine *CDKN2A* screening performed in several European countries [16] and found that approximately half of them could potentially impact on p16 ^INK4a^ post transcriptional regulation, demonstrating that some of them could disrupt IRES activity and influence the translational activity of p16 ^INK4a^. After testing a number of different approaches we suggested a pipeline for the functional analysis that could be applied to characterize any new variant identified in the *CDKN2A* 5`UTR, starting from gene reporter assays based on bicistronic vectors in one melanoma cell line (like WM266-4), integrated with polysomal profiling and p16 ^INK4a^ protein expression studies. The use of cap-independent translation inhibitors was also hypothesized and tested in a separate study to identify an IRES activity in the 5’UTR [17].

When we first found this variant we could consider it as a potential mutation solely on the basis of the allelic frequency in controls and absence in PC patients. While the variants that we previously studied in the 5’UTR were all single nucleotide variants, this variant altered 4 nucleotides. In silico predictions were also uninformative given the localization of the variant (5’UTR) [20].

Interestingly, c.-201_-198delinsCTTT variant was never found in nearly 1,650 of our melanoma cases or in healthy controls [16], nor it is described in public databases. None of the controls from different populations we studied in our previous analysis showed it either. Instead we found it in two unrelated PC cases out of 350 that we studied. No cases of melanoma were observed in these families. We hypothesized that if this variant is functional, it could be specifically associated with PC and thus we studied it using different cell line models, including melanoma, breast cancer and PC cell lines all null for p16 ^INK4a^, as well as normal ductal pancreatic cells.

It is already known that the loss or modification in genes that are part of pathways for specific translational regulatory proteins, can determine an impaired cap-dependent and cap-independent mRNA translation in PC cells[21].

When we studied the c.-201_-198delinsCTTT variant both with monocistronic and bicistronic reporter vectors, we observed no significant differences in cells growing in normal culture conditions. However, when we used Torin1, thereby favoring the IRES activity of the *CDKN2A* 5’UTR, we could appreciate a slight but significant negative impact of the c.-201_-198delinsCTTT variant. Consistently, the variant showed allelic imbalance.

Then we measured the relative allelic representation (wt versus variant) on polysomes, using the subpolysomal fractions as controls and starting from a patient-derived lymphoblastoid cell line that is heterozygous for the variant; the polysomal profile can reveal the relative allelic imbalance in polysomal RNA fractions compared with the sub polysomal RNAs and the c.-201_-198delinsCTTT showed a positive imbalance.

In conclusion, we could classify this variant as a potential mutation with a score of 2, according to the classification proposed in our last study, since it disrupts two of the tested assays [16].

In our cohort we found a 3,5% of germline mutations in the coding region of *CDKN2A* and we found no other potential mutation or rare SNPS in the 5’UTR besides the one here described.

Clearly while the frequency of this variant is too low to be clinically useful (as a useful early detection tool) as it stands, but certainly raises a point regarding this particular variant warranting further investigation in high-risk cohorts. Therefore, our data indicate that sequencing the 5'UTR of CDKN2A should be included in routine screening of PC cases with suspected inherited susceptibility families and that the sequenced region should be extended from current set-up of sequencing to cover the entire 5’UTR region. The identification of this variant only in two unrelated PC cases suggests a specific association with PC and not with melanoma. Recently, frequent noncoding regulatory mutations in PC tissues have been identified by whole genome sequencing studies [22].

As an increasing number of variants of uncertain functional significance (VUS), lying in non-coding regions has been found in cancer susceptibility genes following recent extensive sequencing technological advances. our study may add to the recent investigations of their functional role, and identification of potential mechanism of action [23–26].
